# Detection of Porcine Respirovirus 1 (PRV1) in Poland: Incidence of Co-Infections with Influenza A Virus (IAV) and Porcine Reproductive and Respiratory Syndrome Virus (PRRSV) in Herds with a Respiratory Disease

**DOI:** 10.3390/v14010148

**Published:** 2022-01-14

**Authors:** Aleksandra Woźniak, Piotr Cybulski, Lilla Denes, Gyula Balka, Tomasz Stadejek

**Affiliations:** 1 Department of Pathology and Veterinary Diagnostics, Institute of Veterinary Medicine, Warsaw University of Life Sciences—SGGW, Nowoursynowska 159C, 02-776 Warsaw, Poland; 2 Goodvalley Agro S.A., Dworcowa 25, 77-320 Przechlewo, Poland; piotr.cybulski@goodvalley.com; 3 Department of Pathology, University of Veterinary Medicine, István Str. 2, H-1076 Budapest, Hungary; denes.lilla@univet.hu (L.D.); balka.gyula@univet.hu (G.B.)

**Keywords:** PRV1, PPIV1, IAV, PRRSV, co-infections, nasal swabs

## Abstract

*Porcine respirovirus 1* (PRV1) is also known as porcine parainfluenza virus 1 (PPIV1). The prevalence and the role of PRV1 infections for pig health is largely unknown. In order to assess the PRV1 prevalence in Poland, nasal swabs and oral fluids collected from pigs from 30 farms were examined with RT real-time PCR. Additionally, IAV and PRRSV infection statuses of PRV1-positive samples were examined. The results showed that the virus is highly prevalent (76.7% farms positive) and different patterns of PRV1 circulation in herds with mild–moderate respiratory disease were observed. Co-infections with IAV and PRRSV were infrequent and detected in 8 (23.5%) and 4 (11.8%) out of 34 PRV1-positive nasal swab pools from diseased pens, respectively. In one pen PRV1, IAV, and PRRSV were detected at the same time. Interestingly, PRV1 mean Ct value in samples with co-infections was significantly lower (29.8 ± 3.1) than in samples with a single PRV1 infection (32.5 ± 3.6) (*p* < 0.05), which suggested higher virus replication in these populations. On the other hand, the virus detection in pig populations exhibiting respiratory clinical signs, negative for PRRSV and IAV, suggests that PRV1 should be involved in differential diagnosis of respiratory problems.

## 1. Introduction

*Porcine respirovirus 1* (PRV1), also known as porcine parainfluenza virus 1 (PPIV1), is a member of the family *Paramynxoviridae*. Paramyxoviruses affect humans and a wide range of animals, including livestock species (poultry, cattle, and pigs), and cross-species transmissions were proven [1]. The PRV1 non-segmented negative sense RNA genome (~15 kilobases in size) encodes 6 major proteins: nucleocapsid (N), phosphoprotein (P), matrix (M), fusion (F), haemagglutinin–neuraminidase (H–N), and large (L) proteins (3′-N-P/C/V-M-F-HN-L-5′) [2,3,4,5]. The analysis of F and H–N genes is considered important for molecular epidemiological studies of PRV1, since F and H–N are the major surface proteins, taking part in the binding, entry, and fusion of the virus, and are responsible for neutralizing antibodies induction [2]. The virus is genetically closely related to Sendai virus (SeV) and human parainfluenza virus 1 (HPIV-1), which may suggest its zoonotic potential [6].

*Porcine respirovirus 1* was first identified in Hong Kong in samples from 2008 to 2012 [3] and later in the USA, Chile, Hungary, Germany, and the Netherlands [6,7,8,9]. The role of PRV1 infection for pig health is unknown. The virus was detected in clinically healthy animals, but also in pigs with respiratory clinical signs, including sneezing, coughing, and nasal discharge [2,6,7,8,9,10]. Since PRV1 has been detected in pigs infected with porcine reproductive and respiratory syndrome virus (PRRSV) and influenza A virus (IAV), the virus was proposed to contribute to the porcine respiratory disease complex (PRDC) [5,8,9,10]. On the other hand, the experimental studies carried out in the USA showed no significant clinical respiratory signs in conventional and CD/CD piglets, despite high replication of PRV1 [10], which suggests that the virus alone may not be significantly pathogenic.

The identification of PRV1 in Hungary, Germany and the Netherlands indicates that the virus may be widely spread in Europe. However, no data are available from other important pig producing countries. Therefore, the aim of our study was to assess the prevalence of PRV1 in Poland, with special attention on herds where respiratory disease was observed and the clinical signs suggested the involvement of viral respiratory pathogens.

## 2. Materials and Methods

### 2.1. Study Farms

The study was performed in 2019 and 2020 on 30 commercial Polish pig herds located in different provinces and districts, with different sizes and types of production, belonging to 14 producers (A–N) (Table 1; Supplementary Table S1). In all of the tested pig herds, coughing, sneezing, or nasal discharge in at least one age group were observed, suggestive of viral respiratory infection. Health statuses of herds were reported by veterinary practitioners based on subjective assessments. The samples of nasal swabs (NS) and oral fluids (OF) from clinically affected populations were obtained as a part of a diagnostic investigation in the frame of IAV surveillance program (grant funded by the National Science Centre, Poland, Number: 2018/29/B/NZ7/00257). The samples were collected cross-sectionally from pigs at the age of 5–20 weeks. From each sampled age group, 4–5 nasal swabs were obtained using UTM^®^ system (COPAN Diagnostics Inc., Murrieta, CA, USA). One oral fluid sample was obtained from each sampled pen, as described previously [11]. All samples were delivered to the laboratory, chilled, and then stored at −80 °C.

### 2.2. Detection of PRV1, IAV, and PRRSV in Clinical Samples

Before the nucleic acids extraction, NS were pooled by 4–5, and each pool corresponded to 1 pen of the nursery pigs (5–12-week-old pigs) or fatteners (>12-week-old pigs).

RNA was extracted using QIAamp cador Pathogen Mini Kit (Qiagen, Hilden, Germany), according to the manufacturer’s instruction. Extracted RNA (4 µL) was used for RT real-time PCR with SensiFAST Probe No-ROX One Step Kit Kit (Bioline, London, UK), with primers and a probe targeting a highly conserved region in H–N gene [12]. The amplification was carried out using 6000 Rotor Gene (Qiagen, Hilden, Germany) under the following thermal conditions: 45 °C/10 min, 95 °C/10 min, followed by 40 cycles of 95 °C/15 s, 56 °C/20 s, and 72 °C/20 s. Samples with a cycle threshold (Ct) ≤ 37.0 were considered positive.

The nasal swab pools and the pen-based oral fluids were tested for other viruses involved in respiratory problems in pigs. The IAV detection was performed with *virotype* Influenza A RT-PCR Kit (Indical Bioscience GmbH, Leipzig, Germany) and the PRRSV detection was performed with the previously described method [13].

A farm was considered positive for PRV1, IAV, or PRRSV if at least one sample type, NS or OF, reacted positive in real-time PCR.

### 2.3. Statistical Analyses

Statistical analyses were performed using GraphPad Prism 8 for Windows (GraphPad Software, San Diego, CA, USA). The prevalence of PRV1 in different diagnostic materials (NS and OF) and age groups (nursery pigs and fatteners) was compared using Fischer’s exact test. The comparison of PRV1 Ct values was made using the nonparametric Mann–Whitney test. A two-tailed *p*-value < 0.05 was set as the statistically significant level.

## 3. Results

### 3.1. Detection of PRV1 in Polish Pig Farms

The PRV1 RNA was detected in 23 out of 30 (76.7%) tested farms. In 19 out of 23 farms (82.6%), PRV1 was detected in NS (Table 1). In 1 of these 19 farms (5.3%), the virus was detected only in NS (farm B3) (Table 1). In farms B13, B18, B19, and B20, PRV1 was found only in OF samples (Table 1). In the PRV1-positive farms, the virus was detected in 40 out of 107 (37.4%) of NS pools and 39 out of 99 (39.4%) OF samples (Table 1). The difference between PRV1 detection rates in NS and OF was not statistically significant (*p* > 0.05).

In 7 farms reporting respiratory clinical signs suggestive for viral infection, negative for PRV1, PRRSV (J26, K27, L28, M29, N30), and IAV (J26, K27, N30) were detected, while in farms B24 and B25, none of the tested viruses was detected.

### 3.2. Circulation of PRV1 in Multiplicating and Farrow-to-Finish Farms

Important differences, based on testing NS pools, were observed between the farrow-to-finish farms in the patterns of PRV1 circulation in different age groups (Table 1). In farm E10, PRV1 was detected only in nursery pigs, while in farm F11 the virus was found only in finishers (Table 1). In the rest of farrow-to-finish farms, PRV1 was detected in nursery pigs and finishers. In high health status sow farms, multiplicating replacement gilts internally (B4 and B6), PRV1 was detected only in pigs up to 7 weeks old.

*Porcine respirovirus 1* was detected in 30 out of 64 (46.9%) NS collected from nursery pigs. Significantly lower (*p* < 0.05) prevalence was found in fatteners, where 10 out of 43 (23.3%) of tested NS reacted PRV1-positive. The mean Ct values were similar (*p* > 0.05) in both stages of production: in nursery pigs 31.8 ± 3.2 and in fatteners 30.2 ± 4.9. The virus was the most prevalent in 5-week-old pigs (13 out of 16, 81.3%)—the youngest group tested (Table 1).

### 3.3. Co-Infections of PRV1 with IAV and PRRSV

In 34 out of 40 (85.0%) PRV1-positive pens where ongoing virus infection was identified based on its detection in nasal swabs, mild–moderate respiratory clinical signs were observed (Table 2). The mean PRV1 Ct value in these samples was 31.6 ± 3.7 and no statistically significant differences (*p* > 0.05) were observed in comparison with the pens with no clinical signs (mean Ct = 31.2 ± 5.3) (the pen from which clinical observation was not reported was excluded from the statistical analyses).

The sole PRV1 infection, without the involvement of PRRSV or IAV, was detected in 23 out of 34 (67.6%) of NS pools from pens where respiratory clinical signs were observed (Table 2). In pens where no respiratory signs were observed, PRV1 was also the only virus detected (Table 2).

In some pens where respiratory clinical signs were observed, the co-infections with IAV (8 out of 34—23.5%) and PRRSV (4 out of 34—11.8%) were found. The mean PRV1 Ct value in these samples was significantly lower (29.8 ± 3.1) than in the samples with single PRV1 infection (32.5 ± 3.6) (*p* < 0.05).

## 4. Discussion

Paramyxoviruses are widespread and common in different species. Numerous studies described the PRV1 detection in pigs with different health issues, as well as in animals without any specific clinical signs [5,6,7,8,9,10]. Although the experimental infection of CD/CD pigs indicated that the virus alone may be not pathogenic, its detection in diseased pigs may suggest its accessory role in multifactorial respiratory diseases [10]. In the present study, the cross-sectionally obtained clinical samples from 30 conventional Polish pig farms experiencing mild–moderate respiratory problems, were analyzed with real-time PCR for PRV1 detection. Additionally, IAV and PRRSV infection statuses of PRV1-positive samples were examined.

Our results showed that PRV1 could be widely spread in Poland. As many as 23 of the 30 (76.7%) tested herds from distant provinces and districts were PRV1-positive, based on the detection of the virus in at least one sample, and in 19 farms, PRV1 RNA was detected in nasal swabs, which can be considered as a confirmation of ongoing infection and shedding (Table 1). To our best knowledge, this study is the first one describing PRV1 prevalence in so many farms from a single country. Moreover, in nearly all herds influenza-like respiratory clinical signs were observed. The similar studies performed in Germany and the Netherlands, Hungary, and Chile, involved 26, 22, and 6 farms, respectively [7,8,9]. The presence of PRV1 was confirmed in 42.3% (Germany and the Netherlands), 4.5% (Hungary), and 100.0% (Chile) of farms [7,8,9]. Of these studies, only in the one from Chile the sampling was targeted on herds affected by respiratory disease. So, the results of the Chilean and our studies, as well as the observations from the USA on frequent detection of PRV1 in cases of respiratory disease, may suggest its involvement in the pathogenesis of respiratory diseases. It could also explain much lower prevalence of the virus detected in the samples from randomly selected farms from Hungary.

The previous studies clearly showed that the virus could be detected in many sample types, including lung tissue, nasal and respiratory tract swabs, bronchoalveolar lavage, nasal turbinates, oral fluids, and others [5]. In this study, similar detection rates in NS (40 out of 107, 37.4%) and OF (39 out of 99, 39.4%) were identified (Table 1). Similarly, equal detection rates in these two materials were described in the USA [5]. However, in that study, the prevalence was much higher and PRV1 was detected in 67.6% (175 out of 259) and 64.1% (100 out of 156) of oral fluids and nasal swabs, respectively [5]. In Chile, the PRV1 detection in 62.5% (5 out of 8) oral fluids and 19.0% (23 out of 121) of nasal swabs was described [7]. The explanation of the differences in PRV1 detection rates in NS and OF between this and the previous studies is difficult as the criteria of the farm and samples selection were different or not described [5,7]. It was previously shown that the virus replicates in epithelial cells of upper respiratory tract [6,7]. Although PRV1, as well as any other respiratory virus detection in NS, could be interpreted as ongoing infection in the respiratory tract and be related to some clinical manifestation, the interpretation of the virus detection solely in OF is more difficult. For example, it was shown that IAV RNA could be detected in oral fluids two weeks after nasal shedding ceased [14]. On the other hand, our previous studies showed that PCV2 can be detected in farms and pens where none of the tested pigs were viremic [15]. The finding of PRV1 only in OF samples, in the absence of the virus detection in NS from four tested farms, may be interpreted as the environmental contamination (after the past infections), or as a proof of a very low prevalence of ongoing infection, which the employed protocol of NS collection and testing was not able to detect.

The previous studies provided very little information about PRV1 circulation within farms, and are limited to testing of young pigs only, up to 8 or 11 weeks of age [7,8]. In our study the detailed herd profiles, involving pigs from 5 to >17 weeks of age were performed in 23 farms and different circulation patterns were observed (Table 1). In the farms multiplicating gilts “in-house”, with high biosecurity levels and unified management protocols (B4 and B6), the PRV1 infections were limited to 5–7-week-old pigs only. In smaller, family-owned farms with low internal biosecurity level (e.g., I23, D9, and C8) the virus was detected in NS in nursery pigs and fatteners. It may suggest that, similarly to other viruses, many management factors may affect PRV1 circulation and its impact on the overall health status. Nevertheless, our study supported the previous conclusions that PRV1 infections occur mostly in young animals [8,16]. Consequently, if PRV1 has any impact on pathogenesis of respiratory disease in pigs, the health of weaned pigs is most likely to be affected by the infection.

In some NS samples collected from fatteners high Ct values were observed (Table 1). In farm C8, the infection in 15-week-old pigs was preceded by the infection in 5–7-week-old pigs. The detection of PRV1 in fattening pigs with low Ct values in farms with high biosecurity level (B7 and B3) is difficult to explain. Unfortunately, the data about PRV1-status in weaners placed to these farms was not available. Interestingly, in all fatteners with low Ct values, PRV1 was the only virus detected (Table 2). Thus, more studies are needed to evaluate the significance of PRV1 infection in fatteners.

The effect of PRV1 on pig health remains largely unknown. In this study, the analysis of PRV1 detection in different clinical conditions was assessed. In the vast majority of cases, the virus was detected in pens where respiratory signs were observed (34 out of 40 pens, 85.0%) (Table 2). On the other hand, in 2 pens without apparent respiratory signs, low Ct values (<27.0) were observed, which support previous observations of asymptomatic PRV1 infections (Table 2) [6,7].

*Porcine respirovirus 1* co-infections with IAV and PRRSV were previously described, which may suggest its role as a component of PRDC [5,7,9]. In this study, co-infections with IAV and PRRSV were infrequent and detected in 8 (23.5%) and 4 (11.8%) out of 34 PRV1-positive NS pools, respectively (Table 2). In 1 pen of 7-week-old pigs from farm D9, PRV1, IAV, and PRRSV were all detected (Table 2). Interestingly, the mean PRV1 Ct value of the samples with co-infections was significantly lower (29.8 ± 3.1) than in samples with single PRV1 infection (32.5 ± 3.6) (*p* < 0.05), which suggests higher viral replication in these animals and possible co-stimulatory effects between these viruses.

On the other hand, this study showed the sole PRV1 infection in 23 out of 34 (67.6%) pens where clinical signs were reported (Table 2). It may indicate that, despite previous assumptions, PRV1 alone may cause respiratory disease in conventional pigs or could be a contributing co-factor to different pathogen or other factors induced respiratory issues, not analyzed in the present study.

## 5. Conclusions

In summary, our results show high PRV1 prevalence in Poland, in herds with mild–moderate respiratory disease. The virus detection in diseased pig populations, negative for PRRSV and IAV, suggests that PRV1 should be involved in differential diagnosis, in order to help in the evaluation of the efficacy of control programs involving the vaccination against these viruses.

## Figures and Tables

**Table 1:** The proportion of PRV1-positive NS and OF and summary of PRV1 real-time PCR results in NS expressed as Ct values from 23 PRV1-positive farms. Ct ≤ 37.0 was considered positive.

Farm ID	Type of Production	Ct Values in NS from Different Age Groups							% of PRV1-Positive Samples	
		Nursery Pigs				Fatteners				
		5	7	9	11	13	15	≥17	NS	OF
B4	Multiplicating farms	33.1 ^●^	- ^●^	-	-	-	-	-	14.3	28.6
B6		27.1 ^x^	29.1 ^x^	- ^●^	-	-	-	-	28.6	20.0
B13		-	- ^●^	-	-	-	-	-	-	14.3
A1	Farrow-to-finish farms	-	33.9 ^●^	-	-	-	29.8	-	28.6	14.3
C8		29.1 ^x^	35.5 ^●^		- ^●^	- ^●^	24.7 ^●^		60.0	100.0
D9		23.9 ^x^	29.6 ^●^		30.9 ^●^		31.0 ^●^		100.0	100.0
E10		29.7 ^●^	-	-	-	-	-	- ^●^	14.3	28.6
F11			- ^x^	- ^●^		36.6 ^●^	24.4	36.9	60.0	50.0
G21			31.4 ^●^	35.7	-	-	- ^●^	31.5 ^●^	50.0	50.0
H22		33.3 ^●^		- ^x^		- ^●^	- ^●^		25.0	100.0
I23		30.9	37.0		33.9 ^●^	34.3		-	80.0	14.3
B2	Nursery farms	27.8 ^●^	- ^●^	32.5	-				50.0	50.0
B5		30.6 ^●^	35.3 ^●^	-	36.0 ^●^				75.0	75.0
B12		26.3 ^x^	33.5 ^x^	36.0 ^x^	- ^x^				75.0	not tested
B14		- ^●^	35.6	-	-				25.0	25.0
B15		28.0 ^●^	34.3 ^●^	35.1	- ^●^				75.0	75.0
B16		32.7 ^x^	- ^●^	-	-				25.0	33.3
B17		33.6 ^●^	-	-	-				25.0	25.0
B3	Fattening farms					26.6	-	-	33.3	-
B7						- ^●^	-	24.5 ^●^	33.3	66.6
B18						- ^●^	-	-	-	33.3
B19						- ^●^	-	-	-	33.3
B20						-	-	- ^●^	-	33.3
**% of PRV1-positive** **samples**		**81.3**	**58.8**	**26.7**	**18.8**	**20.0**	**26.7**	**23.1**	**Total NS:** **37.4**	**Total OF:** **39.4**
		**Total:** **46.9**				**Total:** **23.3**				

PRV1—porcine respirovirus 1; NS—nasal swabs pool; OF—oral fluid; gray background—age groups of pigs that were not sampled. -—PRV1-negative samples. ^●^—age groups of pigs with PRV1-positive OF. ^x^—age groups of pigs where OFs were not collected.

**Table 2:** Results of real-time PCR for IAV and PRRSV detection in PRV1-positive nasal swab pools expressed as Ct values. Ct ≤ 37 was considered positive. Health status was reported by veterinary practitioners based on subjective assessments.

Farm ID	Age [Weeks]	Ct Value			Respiratory Signs
		PRV1	IAV	PRRSV	
B2	5	27.8	24.4	-	Coughing, nasal discharge
B2	9	32.5	22.6	-	Coughing, nasal discharge
B5	5	30.6	20.8	-	Coughing
B6	5	27.1	16.6	-	Coughing, sneezing
B6	7	29.1	17.1	-	Coughing, sneezing
B16	5	32.7	20.0	-	Coughing
E10	5	29.7	20.6	-	Coughing, nasal discharge
D9	7	29.6	30.6	31.4	Coughing
D9	5	23.9	-	26.6	Coughing
C8	5	29.1	-	29.9	Coughing
C8	7	35.5	-	32.5	Coughing
A1	7	33.9	-	-	Coughing, nasal discharge
A1	15	29.8	-	-	Coughing, nasal discharge
B4	5	33.1	-	-	Coughing
B5	7	35.3	-	-	Coughing
B5	11	36.0	-	-	Coughing
B12	5	26.3	-	-	Coughing
B12	7	33.5	-	-	Coughing
B14	7	35.6	-	-	Coughing
B15	5	28.0	-	-	Coughing
B15	7	34.3	-	-	Coughing
C8	15	24.7	-	-	Coughing
D9	11	30.9	-	-	Coughing
D9	15	31.0	-	-	Coughing
F11	13	36.6	-	-	Coughing, nasal discharge
F11	15	24.4	-	-	Coughing, nasal discharge
F11	17	36.9	-	-	Coughing, nasal discharge
G21	7	31.4	-	-	Coughing
G21	9	35.7	-	-	Coughing
G21	17	31.5	-	-	Coughing
I23	5	30.9	-	-	Coughing
I23	7	37.0	-	-	Coughing
I23	11	33.9	-	-	Coughing
I23	13	34.3	-	-	Coughing
B3	13	26.6	-	-	None
B7	17	24.5	-	-	None
B12	9	36.0	-	-	None
B15	9	35.1	-	-	None
B17	5	33.6	-	-	None
H22	5	33.3	-	-	Not available

PRV1—porcine respirovirus 1; IAV—influenza A virus; PRRSV—porcine reproductive and respiratory syndrome virus. -—negative samples.

## Data Availability

The data presented in this study are available on request.

## Supplementary Materials

The following supporting information can be downloaded at: https://www.mdpi.com/article/10.3390/v14010148/s1, Table S1: Localization of 30 tested Polish pig farms and proportion of PRV1-positive NS and OF samples.
