# Supplementary material for: Detection of Porcine Respirovirus 1 (PRV1) in Poland: Incidence of Co-Infections with Influenza A Virus (IAV) and Porcine Reproductive and Respiratory Syndrome Virus (PRRSV) in Herds with a Respiratory Disease
Source: Viruses. 2022 Jan 14;14(1):148. doi: 10.3390/v14010148 (PMC8781826; doi:10.3390/v14010148)
Supplement: Supplementary file 1 [file viruses-14-00148-s001.zip › viruses-1525172-supplementary.pdf]

Supplementary Table S1: Localization of 30 tested Polish pig farms and proportion of PRV1-positive NS and OF.

Farm ID	Producer	Type of production	Localization		% of PRV1-positive samples (number of positives/number of tested)	
			Province	District	NS	OF
A1	A	Farrow-to-finish	Lubusz	wschowski	28.6 (2/7)	14.3 (1/7)
B2	B	Nursery	West Pomerania	gryfiński	50.0 (2/4)	50.0 (2/4)
B3	B	Fattening	Greater Poland	złotowski	33.3 (1/3)	- (0/3)
B4	B	Multiplicating farm	Pomerania	człuchowski	14.3 (1/7)	28.6 (2/7)
B5	B	Nursery	Greater Poland	nowotomyski	75.0 (3/4)	75.0 (3/4)
B6	B	Multiplicating farm	Pomerania	człuchowski	28.6 (2/7)	20.0 (1/5)
B7	B	Fattening	Pomerania	człuchowski	33.3 (1/3)	66.6 (2/3)
B12	B	Nursery	Pomerania	człuchowski	75.0 (3/4)	not tested
B13	B	Multiplicating farm	Pomerania	człuchowski	- (0/7)	14.3 (1/7)
B14	B	Nursery	Pomerania	człuchowski	25.0 (1/4)	25.0 (1/4)
B15	B	Nursery	Pomerania	człuchowski	75.0 (3/4)	75.0 (3/4)
B16	B	Nursery	West Pomerania	łobeski	25.0 (1/4)	33.3 (1/3)
B17	B	Nursery	West Pomerania	choszczeński	25.0 (1/4)	25.0 (1/4)
B18	B	Fattening	West Pomerania	białogardzki	- (0/3)	33.3 (1/3)
B19	B	Fattening	West Pomerania	koszaliński	- (0/3)	33.3 (1/3)
B20	B	Fattening	Pomerania	człuchowski	- (0/3)	33.3 (1/3)
B24	B	Multiplicating farm	Kuyavia-Pomerania	sępoleński	- (0/7)	- (0/7)
B25	B	Fattening	West Pomerania	koszaliński	- (0/3)	- (0/3)
C8	C	Farrow-to-finish	Kuyavia-Pomerania	lipnowski	60.0 (3/5)	100.0 (4/4)
D9	D	Farrow-to-finish	Silesia	lubliniecki	100.0 (4/4)	100.0 (3/3)
E10	E	Farrow-to-finish	Lodz	tomaszowski	14.3 (1/7)	28.6 (2/7)
F11	F	Farrow-to-finish	Lodz	tomaszowski	60.0 (3/5)	50.0 (2/4)
G21	G	Farrow-to-finish	Lodz	tomaszowski	50.0 (3/6)	50.0 (3/6)
H22	H	Farrow-to-finish	Mazovia	miński	25.0 (1/4)	100.0 (3/3)
I23	I	Farrow-to-finish	Opole	oleski	80.0 (4/5)	14.3 (1/7)

J26	J	Farrow-to-finish	Lodz	zgierski	- (0/6)	- (0/6)
K27	K	Farrow-to-finish	Mazovia	sokołowski	- (0/7)	- (0/7)
L28	L	Farrow-to-finish	Opole	kluczborski	- (0/5)	- (0/5)
M29	M	Farrow-to-finish	Opole	kluczborski	- (0/5)	- (0/5)
N30	N	Nursery	Lodz	piotrkowski	- (0/4)	- (0/4)

PRV1 – porcine respirovirus 1; NS – nasal swabs pool; OF – oral fluid
- – PRV1-negative samples
